# Epileptic spasms related to neuronal differentiation factor 2 (NEUROD2) mutation respond to combined vigabatrin and high dose prednisolone therapy

**DOI:** 10.1186/s12883-022-02992-9

**Published:** 2022-12-09

**Authors:** Kullasate Sakpichaisakul, Rachata Boonkrongsak, Punjama Lertbutsayanukul, Nareenart Iemwimangsa, Sommon Klumsathian, Bhakbhoom Panthan, Objoon Trachoo

**Affiliations:** 1 Department of Pediatrics, Queen Sirikit National Institute of Child Health, College of Medicine, Rangsit University, Bangkok, 10400 Thailand; 2 Department of Radiology, Neurological Institute of Thailand, Bangkok, Thailand; 3 Centre for Medical Genomics, Faculty of Medicine Ramathibodi Hospital, Mahidol University, Bangkok, 10400 Thailand; 4 Department of Medicine, Faculty of Medicine Ramathibodi Hospital, Mahidol University, 270 Rama 6 Road, Ratchathewi, Bangkok, 10400 Thailand

**Keywords:** Epileptic spasms, NEUROD2, Vigabatrin, Prednisolone, Whole-exome sequencing, Case report

## Abstract

**Background:**

Epileptic spasms are a devastating form of early infantile epileptic encephalopathy (EIEE) with various etiologies. Early diagnosis and a shorter lead time to treatment are crucial to stop the seizures and optimize the neurodevelopmental outcome. Genetic testing has become an integral part of epilepsy care that directly guides management and family planning and discovers new targeted treatments. Neuronal differentiation Factor 2 (NEUROD2) variants have recently been a cause of neurodevelopmental disorders (NDDs) and EIEEs with distinctive features. However, there is limited information about the clinical and electroencephalographic response of epileptic spasm treatment in NEUROD2-related NDD syndrome.

**Case presentation:**

We report a female patient of Southeast Asian ethnicity with global developmental delay and epileptic spasms commencing in the first few months of life. A novel de novo heterozygous pathogenic *NEUROD2* variant, p. E130Q, was subsequently identified by whole-exome sequencing. Electroencephalogram before treatment showed multifocal independent spikes predominantly in both posterior head regions and demonstrated marked improvement following combined vigabatrin and high-dose prednisolone treatment. However, multiple courses of relapse occurred after weaning off the antiseizure medication.

**Conclusions:**

We propose that epileptic spasms related to de novo *NEUROD2* pathogenic variant respond well to combined vigabatrin and high-dose prednisolone therapy. These findings may imply the benefit of using combination therapy to treat epileptic spasms in NEUROD2-related NDD syndrome.

## Background

Epileptic spasms (ESs) are the most common form of early infantile epileptic encephalopathy (EIEE). ES is typically accompanied by a hypsarrhythmia pattern or variants on electroencephalography (EEG) and developmental delay. Treatment options are vigabatrin, adrenocorticotropic hormone (ACTH), high-dose prednisolone, or a combination of steroid and vigabatrin therapy [1]. The proven underlying etiologies of ES accounted for the majority of patients through clinical assessment, neuroimaging, and genetic testing. However, in one-third of children, no etiology was identified [2]. Advances in genetic technology unravel many genetic anomalies that disrupt fundamental processes in the developing brain and affect clinical epilepsy care [3].

Neuronal differentiation Factor 2 (NEUROD2) (OMIM# 601725) is a transcription factor in the basic helix-loop-helix family that plays a critical role in nervous system development and function [4]. Specifically, NEUROD2 regulates neuronal migration and differentiation and balances synaptic neurotransmission by promoting inhibitory synaptic drive and decreasing cell-intrinsic neuronal excitability [4, 5]. Recently, NEUROD2 mutation has been shown to be the causative of neurodevelopmental disorders (NDDs) with core clinical features including intellectual disability, autism spectrum disorders, and speech disturbance [6–8]. However, there are limited data about the treatment course of ES with combined vigabatrin and high-dose prednisolone in the NEUROD2-related NDD syndrome. Here, we present a patient with ES and developmental delay related to a de novo *NEUROD2* genomic variant with detailed clinical characteristics, serial EEGs, neuroimaging findings, and response to treatment.

## Case presentation

According to the Ethics Committee of Queen Sirikit National Institute of Child Health, case report consent forms were obtained. The patient was the first child of nonconsanguineous parents with uncomplicated pregnancy. She was born at full term via cesarean section with a birth weight of 4500 g. Within 3 months of life, her parents noticed that she had developmental delay by not making good eye contact and no social smile. At 5 months of age, she developed the first seizure characterized by flexion of her arms, legs, and body every 5–6 seconds up to 10–15 times per cluster. Vigabatrin, 50 mg/kg/day, combined with high-dose prednisolone, 60 mg/day, was started, and cessation of spasms was obtained within 24 hours. Prednisolone was tapered within 28 days, vigabatrin was continued for 6 months, and then the patient was gradually weaned. She had been seizure-free with a period of no antiseizure medication (ASM) for 6 months.

**Fig. 1:**
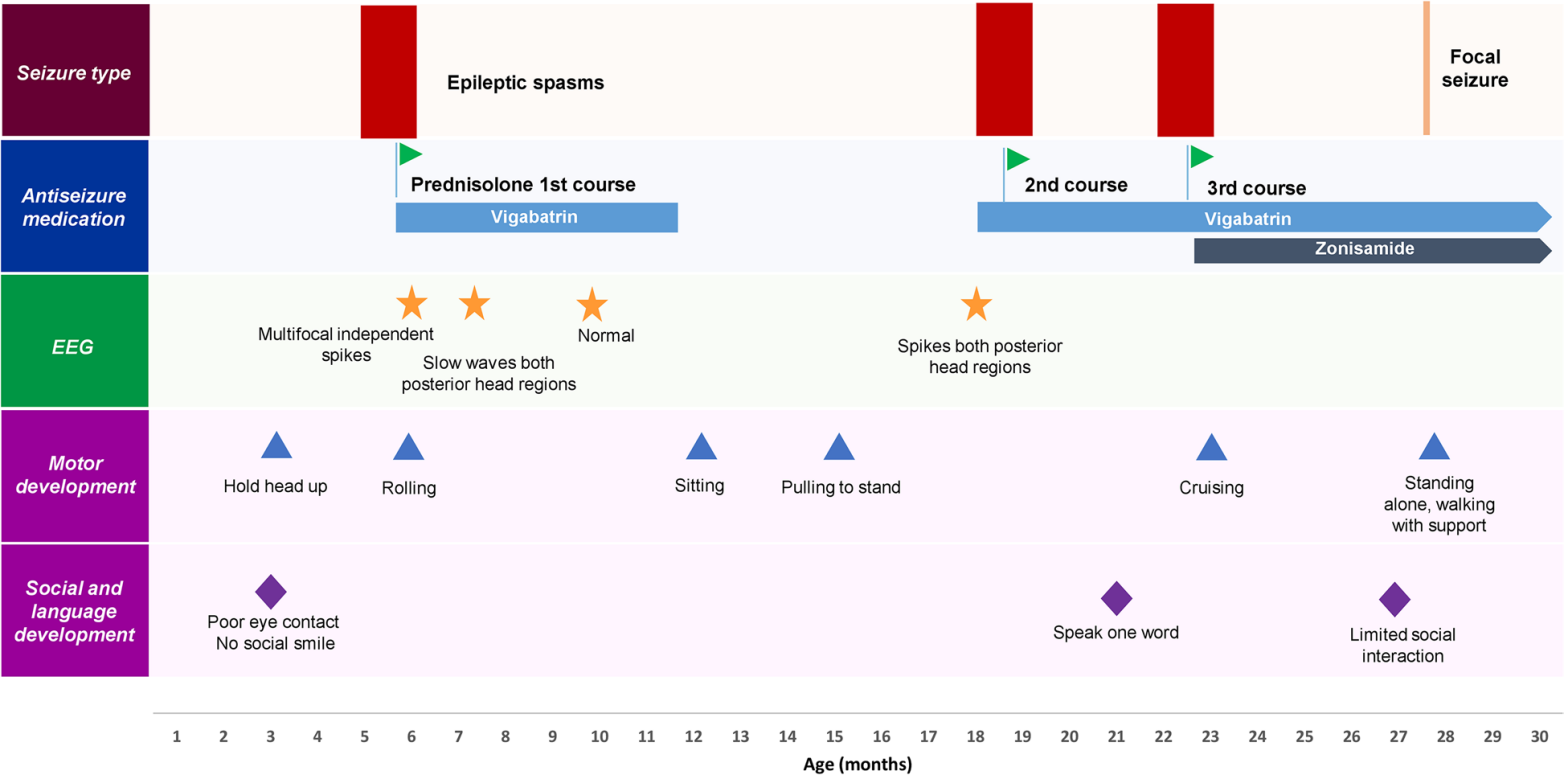
A schematic representation of the seizure onset, therapy, electroencephalography (EEG), and developmental milestones in the reported case

The first relapse of clinical spasms occurred at 17 months of age. Vigabatrin alone was restarted and titrated up to 150 mg/kg/day but did not achieve a cessation of spasms. Subsequently, the 2nd course of high-dose prednisolone was combined with vigabatrin, which led to a cessation of spasms. However, she relapsed of spasms again at 20 months of age after prednisolone stopped. Therefore, the 3rd course of prednisolone combined with vigabatrin was introduced, and clinical spasms disappeared. At that time, zonisamide was prescribed alongside vigabatrin to prevent relapse. Regaining in development was noted since the cessation of spasms was obtained. She developed focal seizures characterized by episodes of screaming with limb automatism at 2 years of age that were well controlled after adjusting for ASMs. At the most recent physical examination at 27 months of age, anisocoria and esotropia were noted. Receptive language and social domains are markedly delayed compared to motor domains. The seizure onset, ASMs, EEG evolution, and developmental milestones progression were illustrated in Fig. 1.

**Fig. 2:**
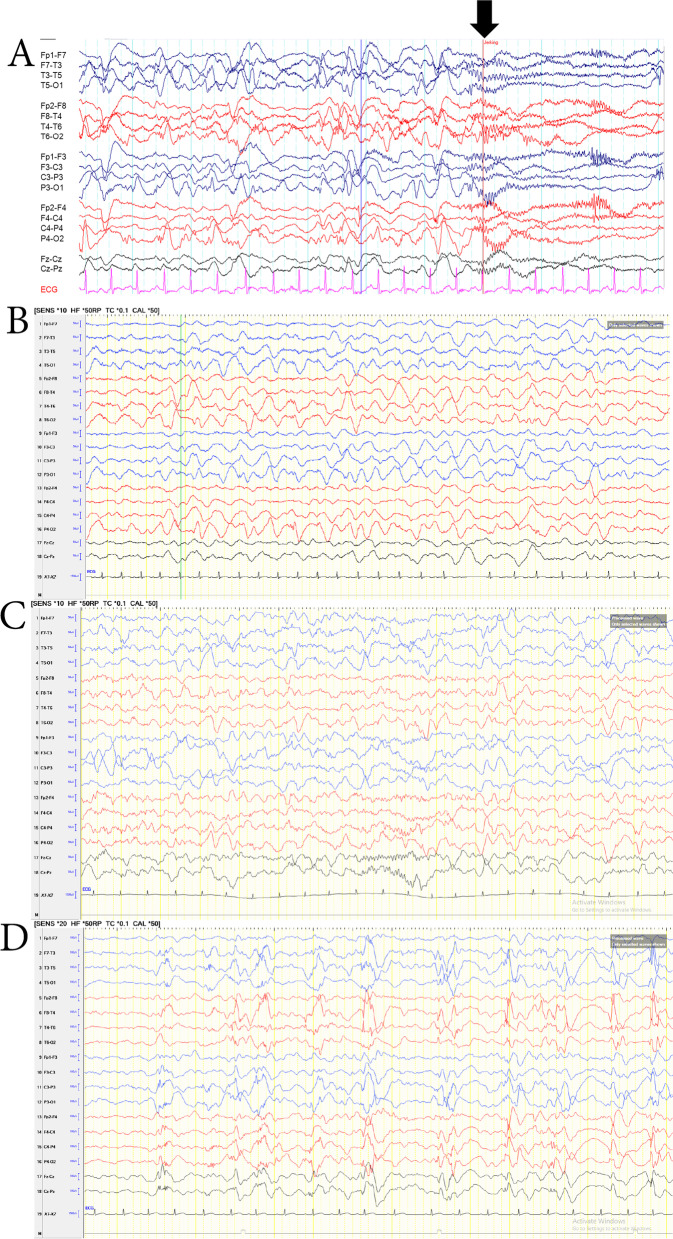
The evolution of electroencephalography (EEG) of the patient. **A** Initial EEG consisted of high amplitude multifocal spikes > 50% with bilateral posterior head regions predominance. Ictal pattern characterized by high voltage slow waves superimposed with fast activity (arrow); **B** the EEG on Day 14 after treatment shows high amplitude slow waves intermixed with spikes in the bilateral posterior head quadrant regions and decreased epileptiform discharges; **C** the EEG three months after treatment is normal, with well-formed sleep spindles seen; **D** the EEG at two years of age reveals high amplitude spikes in the bilateral parieto-temporal head regions

The first EEG before treatment showed widespread multifocal independent spikes predominantly in both posterior head regions. The EEG at Day 14 after treatment demonstrated improvement characterized by decreased epileptiform discharges. Normalization of EEG was noted 3 months after treatment. Serial EEG assessment at 16 months showed reappearance of spikes in the bilateral posterior head regions before clinical spasm relapse. Her recent EEG at 2 years of age still showed spikes in the bilateral posterior head regions (Fig. 2).

**Fig. 3:**
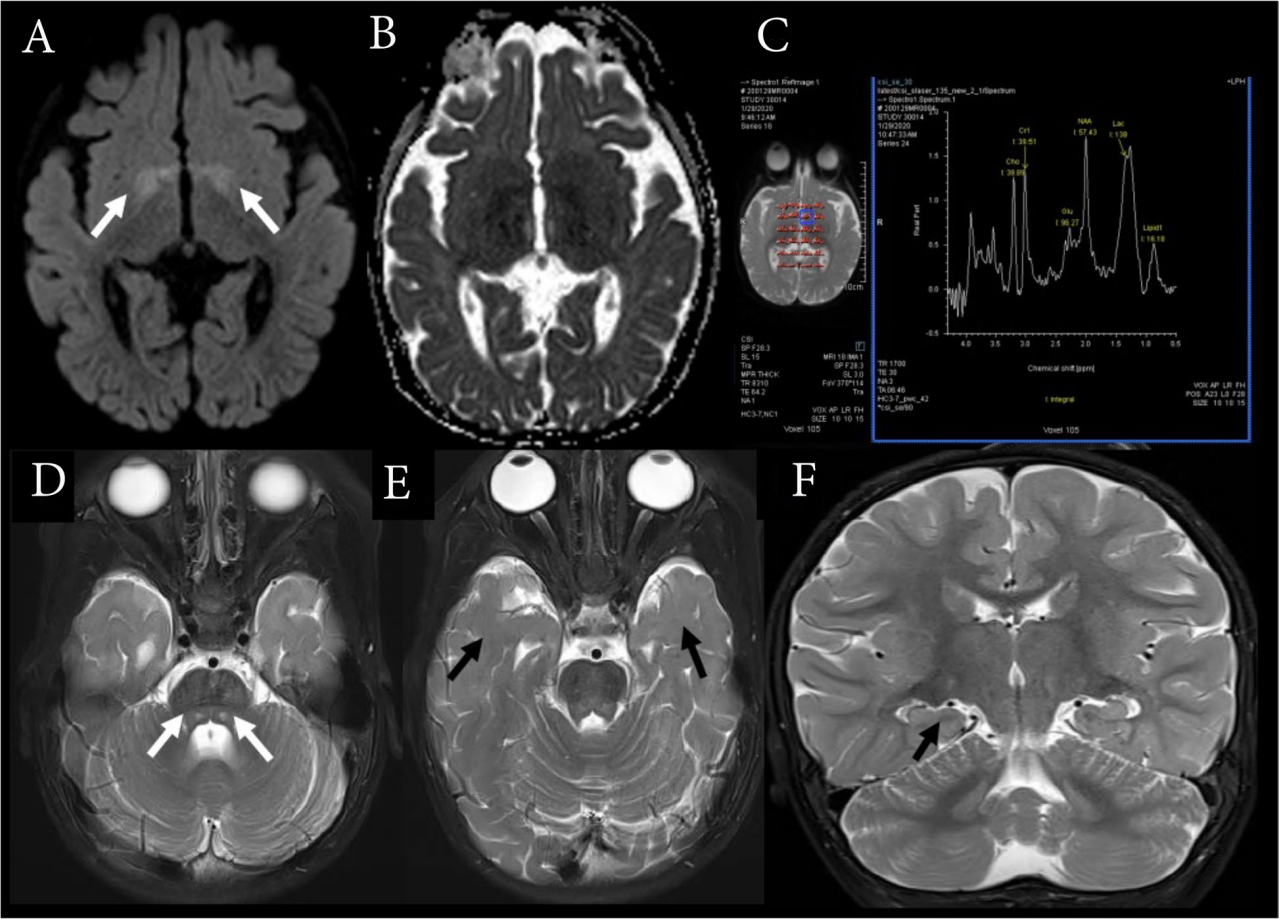
Magnetic resonance imaging (MRI) of the brain was performed at six months when the patient was on vigabatrin and prednisolone. (**A**) axial diffusion-weighted imaging (DWI); (**B**) apparent diffusion coefficient (ADC) mapping displaying a restricted diffusion at the bilateral globi pallidi; (**C**) multivoxel magnetic resonance spectroscopy (MRS) with a short echo time (30 milliseconds) at the left globus pallidus displaying a decrease of N-acetyl aspartate (NAA) and the presence of lactate peak. Brain MRI, T2-weighted images at 26 months of age, shows small hyper signal lesions at (**D**) bilateral dorsal pons; (**E**) delayed myelination for age; and (**F**) small right hippocampus

Magnetic resonance imaging (MRI) was performed at 6 months of age and revealed mild T2 hyperintensities and restricted diffusion at the bilateral globi pallidi. MR spectroscopy with a short echo time (30 milliseconds) displayed decreased N-acetylaspartate and the presence of a lactate peak. Metabolic investigations, including blood lactate, plasma amino acid, and urine organic acid, were normal. The second MRI was performed at 2 years of age and revealed symmetrical hyperintensities in the bilateral central tegmental tracts (CTT) at the dorsal pons on both T2 and fluid-attenuation inversion recovery images. There was delayed myelination of subcortical white matter in the bilateral frontal and temporal lobes and a small right hippocampus. The disappearance of the previous globus pallidus lesions on the first MRI was noted (Fig. 3).

Subsequent searching for the genetic etiology of this case was done by trio whole-exome sequencing using the leukocyte DNAs obtained from the patient and both parents. Next-generation sequencing was performed by SureSelect Human All Exon V7 (Agilent, Inc**.)** on Illumina Technology (Illumina, Inc)**.** The DNA sequences were assembled, aligned against reference gene sequences based on human genome build GRCh37**/**UCSC hg19, and analyzed for sequence variants using GATK Pipeline for calling variants (Broad Institute). A total of 161,808 variants in the family were detected, and 12,152 were related to epilepsy. Afterwards, bioinformatic analysis was performed using multiple modes of Mendelian inheritance, including autosomal dominant, autosomal recessive (homozygosity and compound heterozygosity) and X-linked manners. Finally, a de novo pathogenic variant in *NEUROD2* was revealed. The discovered variant was identified as heterozygous c.388G > C at the mRNA level (NM_006160.3) or p. E130Q at the protein level, which was classified as pathogenic based on the criteria provided by the American College of Medical Genetics and Genomics [9].

## Discussion and conclusions

This report presents the clinical characteristics, serial EEG and MRI findings, and developmental status of a patient with ES related to a de novo pathogenic variant in *NEUROD2*. Although cessation of clinical ES was achieved with a combination of vigabatrin and high-dose prednisolone treatment, she had multiple relapses and global developmental delay. Our patient phenotype shared similar clinical onset of ES with a milder developmental delay than the previously reported two patients with a similar p. E130Q pathogenic variant [7, 8] (Table 1).

**Table 1:** Clinical characteristics of patients reported with similar *NEUROD2* variants

Characteristics	Sakpichaisakul et al., 2022 (this study)	Sega et al., 2019	Runge et al., 2021
*NEUROD2* variant	c.388G > C, *p*. E130Q	c.388G > C, *p*. E130Q	c.388G > C, *p*. E130Q
Inheritance	De novo	De novo	De novo
Sex	Female	Female	Male
Birth weight (g), length (cm), head circumference (cm)	4500, 55, 36	3175, NA, NA	3175, 51.4, 31.8
Age at last examination	27 months	5 years	21 months
Weight, height, and head circumference at last examination	50th -75th, 50th, <3rd percentile	3rd –10th, 3rd, <3rd percentile	9.48 kg, 81.5 cm, 43.5 cm (< 1 percentile)
Failure to thrive	No	Yes	No
Onset of developmental delay (months)	2–4	2–4	NA
Onset of seizure (months)	5	5	4
Seizure type	Epileptic spasms	Epileptic spasms	Epileptic spasms
Initial EEG features	Multifocal epileptiform discharges	Hypsarrhythmia	NA
ASM previously tried	Vigabatrin, prednisolone, zonisamide	ACTH, prednisolone, pyridoxine, vigabatrin	NA
Seizure freedom	Yes, seizure free since the age of 22 months, currently on vigabatrin and zonisamide	Seizure free since the age of 2.5 years. Started on the ketogenic diet at 16 months, discontinued at 3 years	NA
Last EEG after being seizure-free	Intermittent epileptiform discharges over the bilateral posterior head regions.	Intermittent epileptiform discharges over the left temporal region and excessive slow waves over bilateral occipital regions during sleep, no electrographic seizures.	NA
Brain MRI features	At 6 months: increased T2/FLAIR signal intensity with restricted diffusion in bilateral globus pallidus. MRS: decreased NAA and presence of small lactate peak At 24 months: symmetric hypersignal T2/FLAIR lesions without restricted diffusion in the central tegmental tract, delayed myelination in bilateral fronto-temporal lobes, and small right hippocampus.	At 6 months: delayed myelination and thin corpus callosum. At 12 months: asymmetrical increased T2 signal intensity in bilateral parietal white matter, thin corpus callosum, prominent frontotemporal CSF spaces, improved myelination. At 3.5 years: bilateral increased T2 signal intensities in putamina, parietal periventricular white matter, diffuse thinning of corpus callosum, normal myelination.	NA
Walking delay	Yes, currently stands alone, walks with support	Yes, still needs a walker	Most likely, yes (not yet)
Intellectual disability	Yes	Yes, severe cognitive dysfunction, is not able to understand simple commands	Yes
Speech disturbance	Yes, speaks one word	Yes, 5 single words only, no sentences	Yes, delayed
Autistic features/behavioral disorder	Yes, limited social interaction and repetitive behavioral patterns	NA	ASD
ADHD/aggressiveness	No	Yes, hyperkinetic movements	No
Physical exam features	Bilateral esotropia, anisocoria, repetitive hand and mouth movements	Bilateral esotropia, hyperkinetic movements	Microcephaly, hypotonia, global developmental delay, feeding difficulties, constipation

*Abbreviations*: *ADHD* Attention-deficit hyperactive disorder, *ASM* Antiseizure medications, *ASD* Autism spectrum disorders, *CBD* Cannabidiol, *FLAIR* Fluid-attenuated inversion recovery, *KD* Ketogenic diet, *NAA* N-acetylaspartate, *NA* Not applicable

Early diagnosis and aggressive treatment in ES optimize both seizure and neurodevelopmental outcomes. ACTH is preferred as the first-line treatment for ES related to non-tuberous sclerosis complex in many well-developed tertiary-care centers. However, ACTH is costly and not commercially available in our country. Therefore, vigabatrin alone is our professional society’s first recommended therapy in this situation. Recent evidence has demonstrated that combination steroid therapy with vigabatrin is more effective than steroids alone, which leads us to initiate early combined therapy [1]. In our patient, vigabatrin alone, which inhibits γ-aminobutyric acid (GABA) transaminase, was insufficient to cease spasms. Combining prednisolone with vigabatrin treatment elicited a more satisfactory outcome. Theoretically, the pathogenesis of ES is the excess of corticotropin-releasing hormone (CRH) synthesis in response to stress. CRH increases excitability in the amygdala-hippocampal limbic circuit and results in developing ES. The probable mechanism of prednisolone is the suppressive effect of CRH [10].

Mainly, *NEUROD2* mutation disrupts cortical neurons’ inhibition/excitation balance and impairs glutamatergic and GABAergic transmission, particularly in the amygdala and hippocampus [5, 11]. The mutation is potential to activate the intrinsic response of the developing brain to stress. We speculate that the CRH-excess theory possibly explained the response to treatment from prednisolone in our patient. Furthermore, vigabatrin and methylprednisolone treatment effectively suppressed spasms in the betamethasone-N-Methyl-D-Aspartic Acid (NMDA)-induced spasms model in rats [12].

However, the response to treatment in this patient is brutal to determine whether this resulted from a synergistic effect of combination treatment or responded through reduced concentrations of the pro-epileptogenic neuropeptide corticotropin-releasing hormone of the steroid itself [1, 10]. NEUROD2 function is evidence to balance synaptic neurotransmission and intrinsic excitability of cortical pyramidal neurons [5]. Although this is an observational finding, it is potentially clinically relevant, as clinicians may consider early combination treatment for patients with *NEUROD2* variant-related EIEEs. The multiple relapse courses of epileptic spasms in this patient are not yet fully understood and may be attributed to prednisolone treatment or the natural course of *NEUROD2* disorders.

Evaluation of EEG is critical for the diagnosis of ES and posttreatment response. We demonstrated EEG evolution in the first course of treatment (Fig. 2). High amplitude slow waves intermixed with spikes located in the bilateral posterior head regions were noted in our patient before clinical spasm relapsed and persisted until the recent EEG at 2 years of age. The characteristics of EEG patterns in NEUROD2-related EIEEs have not been well elucidated, and further study is warranted. Hence, serial EEG assessment may be a helpful monitoring tool in treating epileptic spasms related to *NEUROD2* mutation.

Previously reported MRI findings in *NEUROD2* mutation patients did not show structural cortical malformations that cause severe epilepsy. The first MRI in our patient displayed no parenchymal abnormality apart from vigabatrin-associated MRI signal changes [13]. Interestingly, at 2 years of age, an MRI revealed symmetrical CTT lesions, delayed myelination, and a small right hippocampus. CTT lesions were associated with various neurological conditions and high prevalence among children with epileptic spasms [14]. It remains unclear whether this MRI finding was pathologic or a physiologic maturation-related process [14]. Delayed myelination has been previously reported in a patient with similar variants and subsequent normal myelination at 3.5 years (Table 1; Sega et al.). A small hippocampus may be related to fewer surviving neurons in the hippocampus, as evidenced by higher rates of apoptosis and smaller brains in NEUROD2-absent mice [4]. Nevertheless, the distinct MRI pattern in the *NEUROD2* variant is not yet known.

*NEUROD2*-related NDD syndrome has been recently demonstrated that forebrain excitatory neurons are the cellular origin of the main neurobehavioral symptoms. Despite multiple relapses of epileptic spasms, our patient had milder developmental delay than the similar mutation in the previously reported patient (Table 1; Sega et al.) and insufficient data to compare with the other patient (Table 1; Runge et al.). These findings can be the effect of treatment response with early combination therapy or genotypic-related function. Unfortunately, functional testing was not available in this study. However, a previous study displayed a depletion of *NEUROD2* expression following CRISPR/Cas9-mediated genome editing, inducing spontaneous seizures in tadpoles [6]. Thus far, a future perspective in this field is to study additional promising data aiming for therapeutic discovery, i.e., in vitro studies in human inducible pluripotent stem cells and in vivo studies in mammals.

Our report presented data showing that the *NEUROD2* pathogenic variant occurred de novo by whole-exome sequencing. Relying on a genetic testing result, a recurrent risk in the family is less than 1%; therefore, the prenatal genetic diagnosis would be comprehensively discussed and recommended to the parents for their conception plan.

In conclusion, we provide the electroclinical characteristics of epileptic spasms with multiple relapses responding to combining vigabatrin and high-dose prednisolone therapy in a patient with *NEUROD*-related NDD syndrome. Identification of further patients will help elucidate the spectrum of genotype-phenotype correlations.

## Data Availability

The datasets used and/or analyzed during the current study are available from the GenBank repository, NC_060941.

## Abbreviations

ACTH: Adrenocorticotropic hormone
ADC: Apparent diffusion coefficient
ADHD: Attention-deficit hyperactive disorder
ASD: Autism spectrum disorder
ASM: Antiseizure medication
CBD: Cannabidiol
CRH: Corticotropin-releasing hormone
CTT: Central tegmental tract
DWI: Diffusion-weighted imaging
EEG: Electroencephalography
EIEE: Early infantile epileptic encephalopathy
ES: Epileptic spasm
FLAIR: Fluid-attenuated inversion recovery
GABA: γ-aminobutyric acid
KD: Ketogenic diet
MRI: Magnetic resonance imaging
MRS: Magnetic resonance spectroscopy
NA: Not applicable
NDD: Neurodevelopmental disorder
NEUROD2: Neuronal Differentiation Factor 2
NAA: N-acetyl aspartate
NMDA: N-methyl-D-aspartic acid
